# Supplementary material for: Content-rich biological network constructed by mining PubMed abstracts
Source: BMC Bioinformatics. 2004 Oct 8;5:147. doi: 10.1186/1471-2105-5-147 (PMC528731; doi:10.1186/1471-2105-5-147)
Supplement: Additional File 2 — The original results of the above study (non-essential files are deleted to keep the file size under the limit set by BMC bioinformatics). [file 1471-2105-5-147-S2.bz2 › chilibotAdditionalFile2/dip20/679ID9514722E11088/html/MAUB_MAUA.html]

 


 **MAUB** and **MAUA** 
  
Found 74 abstracts in PubMed, retrieved 20.  
 

 What does Google say? 
 PDF only 
| .edu only 

---

**Interactive relationship** (e.g. stimulation, inhibition, etc)

**Inhibitory relationship**- The alpha D180R mutation also eliminated the ionic strength dependence of the reaction of MADH  [ **MAUA** ]   [ **MAUB** ]  with amicyanin that is observed with wild type MADH  [ **MAUA** ]   [ **MAUB** ] .  Ref: 10913294 Biochemistry, 2000
- Monovalent cations are known to affect the spectral properties of MADH  [ **MAUA** ]   [ **MAUB** ]  and to influence the rate of the gated electron transfer ET reaction from substrate reduced MADH  [ **MAUA** ]   [ **MAUB** ]  to amicyanin.  Ref: 11591147 Biochemistry, 2001
- alphaF55A MADH  [ **MAUA** ]   [ **MAUB** ]  exhibits an increased K d for cation dependent spectral changes and a decreased K d for cation dependent stimulation of the rate of gated ET from N quinol MADH  [ **MAUA** ]   [ **MAUB** ]  to amicyanin.  Ref: 12686138 Biochim Biophys Acta, 2003
**Neutral relationship**- The crystal structure of this protein complex is known, and the importance of certain residues on amicyanin in its interaction with MADH  [ **MAUA** ]   [ **MAUB** ]  has been demonstrated by site directed mutagenesis.  Ref: 10913294 Biochemistry, 2000
- The crystal structure of MADH  [ **MAUA** ]   [ **MAUB** ]  reveals that each of these residues participates in hydrogen bonding interactions with other active site residues, TTQ or water.  Ref: 11707614 Protein Eng, 2001
- Phylogenetic analysis of amine dehydrogenases MADH  [ **MAUA** ]   [ **MAUB** ]  and AADH indicated that AADH and MADH  [ **MAUA** ]   [ **MAUB** ]  evolutionarily diverged before separation of proteobacteria into existing subclasses.  Ref: 11495996 Microbiology, 2001
- Another site directed mutant, alpha F55E MADH  [ **MAUA** ]   [ **MAUB** ] , exhibited cation binding properties that were the same as those of the native enzyme, indicating that interactions with the carboxylate of Glu can effectively replace the cation pi interactions with Phe in stabilizing monovalent cation binding to the distal site.  Ref: 11591147 Biochemistry, 2001
- This alphaF55A MADH  [ **MAUA** ]   [ **MAUB** ]  exhibits a 400 fold lower Km value for histamine than does native MADH  [ **MAUA** ]   [ **MAUB** ]  when assayed in solution.  Ref: 11924976 Anal Chem, 2002
- For each mutant, the majority of the protein that was isolated was inactive and exhibited weaker subunit subunit interactions than native MADH  [ **MAUA** ]   [ **MAUB** ] .  Ref: 12641453 Biochemistry, 2003
- Very low levels of active MADH  [ **MAUA** ]   [ **MAUB** ]  were produced by cells expressing D32N, Y119F, Y119E and Y119K MADH  [ **MAUA** ]   [ **MAUB** ]  mutants.  Ref: 11707614 Protein Eng, 2001
- The biogenesis of the quinone cofactor tryptophan tryptophylquinone TTQ in methylamine dehydrogenase MADH  [ **MAUA** ]   [ **MAUB** ]  involves the post translational modification of two constitutive Trp residues Trp beta 57 and Trp beta 108 in Paracoccus denitrificans MADH  [ **MAUA** ]   [ **MAUB** ] .  Ref: 12641453 Biochemistry, 2003
- This mutation had no effect on the parameters for methylamine oxidation by MADH  [ **MAUA** ]   [ **MAUB** ] , but significantly affected its interaction with amicyanin.  Ref: 10913294 Biochemistry, 2000
- The alphaF55A MADH  [ **MAUA** ]   [ **MAUB** ]  immobilized on the electrode exhibited Michaelis Menten behavior in response to varied concentrations of histamine with an approximately 3 fold lower Km value than that exhibited by the immobilized native MADH  [ **MAUA** ]   [ **MAUB** ] .  Ref: 11924976 Anal Chem, 2002
- The detection limit for the native MADH  [ **MAUA** ]   [ **MAUB** ]  PPy sensor was approximately 20 microM while the alphaF55A MADH  [ **MAUA** ]   [ **MAUB** ]  PPy sensor exhibited a detection limit of approximately 5 microM, a 4 fold increase compared to the native MADH  [ **MAUA** ]   [ **MAUB** ]  PPy sensor.  Ref: 11924976 Anal Chem, 2002

**Non-interactive relationship** (e.g. studied together, co-existance, homology, etc.)

- To further modulate the substrate specificity of MADH  [ **MAUA** ]   [ **MAUB** ] , the side chain of alphaPhe 55 was repositioned by site directed mutagenesis of residue betaIle 107.  Ref: 11733518 J Biol Chem, 2002
- In contrast, with alphaF55E MADH  [ **MAUA** ]   [ **MAUB** ]  cross linking of subunits is observed.  Ref: 12062431 FEBS Lett, 2002
- Our observations illustrate the different modes of H transfer in MADH  [ **MAUA** ]   [ **MAUB** ]  and AADH with fast and slow substrates and highlight the importance of barrier shape in determining reaction rate.  Ref: 11087744 J Biol Chem, 2001
- Preliminary results obtained with MADH  [ **MAUA** ]   [ **MAUB** ]  enzyme electrodes demonstrate the potential for quinoprotein based biosensors.  Ref: 10721104 Adv Exp Med Biol, 1999
- MADH  [ **MAUA** ]   [ **MAUB** ]  converts primary amines to their corresponding aldehydes plus ammonia.  Ref: 10721104 Adv Exp Med Biol, 1999
- Site directed mutagenesis of either alphaPhe 55 or betaIle 107 a residue in the beta subunit which interacts with alphaPhe 55 converts MADH  [ **MAUA** ]   [ **MAUB** ]  into enzymes with specificities for long chain amines, amylamine or propylamine.  Ref: 12686138 Biochim Biophys Acta, 2003
- Methylamine dehydrogenase MADH  [ **MAUA** ]   [ **MAUB** ]  may be immobilized in a polypyrrole PPy film on an electrode surface and used as an amperometric sensor for the determination of histamine.  Ref: 11924976 Anal Chem, 2002
- Interestingly, the inverse mutation pair of alpha D180R MADH  [ **MAUA** ]   [ **MAUB** ]  and R99D amicyanin did not restore the favorable salt bridge, but instead disrupted complex formation much more severely than did either individual mutation.  Ref: 10913294 Biochemistry, 2000
- The K m for 0 diaminodecane is 11 microM, compared to 1.2 mM for wild type MADH  [ **MAUA** ]   [ **MAUB** ] .  Ref: 10985763 Biochemistry, 2000
- Methylamine dehydrogenase MADH  [ **MAUA** ]   [ **MAUB** ]  is a tryptophan tryptophylquinone TTQ dependent enzyme that catalyzes the oxidative deamination of primary amines.  Ref: 11591147 Biochemistry, 2001
- The resulting inactivation is accompanied by the formation of a covalent cross link between the alpha and beta subunits of MADH  [ **MAUA** ]   [ **MAUB** ] .  Ref: 12062431 FEBS Lett, 2002
- These properties are in marked contrast with those of MADH  [ **MAUA** ]   [ **MAUB** ] , which shows high specificity to amicyanin.  Ref: 12686147 Biochim Biophys Acta, 2003
- This paper reports the first site directed mutagenesis of MADH  [ **MAUA** ]   [ **MAUB** ] , as well as the construction, heterologous expression, and characterization of a six His tagged MADH  [ **MAUA** ]   [ **MAUB** ] . alpha Asp180 of MADH  [ **MAUA** ]   [ **MAUB** ]  was converted to arginine to examine the effect on complex formation with native and mutant amicyanins.  Ref: 10913294 Biochemistry, 2000
- Conversion of alphaPhe55 to alanine dramatically alters the substrate preference of MADH  [ **MAUA** ]   [ **MAUB** ] .  Ref: 10985763 Biochemistry, 2000
- It is now shown that the alphaF55A mutation also increases the rate of the true electron transfer ET reaction from O quinol MADH  [ **MAUA** ]   [ **MAUB** ]  to amicyanin.  Ref: 12437349 Biochemistry, 2002
- The biosynthesis of methylamine dehydrogenase MADH  [ **MAUA** ]   [ **MAUB** ]  from Paracoccus denitrificans requires four genes in addition to those that encode the two structural protein subunits.  Ref: 12809487 Biochemistry, 2003
- One of these, mauG, exhibits sequence similarity to diheme cytochrome c peroxidases and is required for the synthesis of the tryptophan tryptophylquinone TTQ prosthetic group of MADH  [ **MAUA** ]   [ **MAUB** ] .  Ref: 12809487 Biochemistry, 2003
- The crystal structure of alphaF55A MADH  [ **MAUA** ]   [ **MAUB** ]  in complex with its electron acceptors, amicyanin and cytochrome c 551i, has been determined.  Ref: 12437349 Biochemistry, 2002
- This MADH  [ **MAUA** ]   [ **MAUB** ]  PPy sensor exhibited a response time of less than 3 s.  Ref: 10845365 Anal Chem, 2000
- betaI107V MADH  [ **MAUA** ]   [ **MAUB** ]  exhibits a strong preference for propylamine, and betaI107N MADH  [ **MAUA** ]   [ **MAUB** ]  exhibits a preference for 1 aminopentane.  Ref: 11733518 J Biol Chem, 2002
- Bond cleavage by MADH  [ **MAUA** ]   [ **MAUB** ]  using a slow substrate ethanolamine occurs with an inflated KIE 14.7 0.2 at 25 degrees C.  Ref: 11087744 J Biol Chem, 2001
- Methylamine dehydrogenase MADH  [ **MAUA** ]   [ **MAUB** ]  is produced by a range of gram negative methylotrophic and autotrophic bacteria, and allows the organisms to utilise methylamine as the sole source of carbon.  Ref: 12686162 Biochim Biophys Acta, 2003
- A stable binary MADH  [ **MAUA** ]   [ **MAUB** ]  amicyanin complex can be formed, and its crystal structure has been solved to 2.5 A resolution by Chen et al.  Ref: 12686162 Biochim Biophys Acta, 2003
- In this study, site directed mutagenesis of MADH  [ **MAUA** ]   [ **MAUB** ] , kinetic data, and thermodynamic analysis are used to probe the molecular basis for stabilization of the protein complex by an interprotein salt bridge between Arg99 of amicyanin and Asp180 of the alpha subunit of MADH  [ **MAUA** ]   [ **MAUB** ] .  Ref: 10913294 Biochemistry, 2000
- Site directed mutagenesis studies have revealed several roles for this residue in catalysis and electron transfer ET by MADH  [ **MAUA** ]   [ **MAUB** ] .  Ref: 12686138 Biochim Biophys Acta, 2003
- Tryptophan tryptophylquinone TTQ, the cofactor of methylamine dehydrogenase MADH  [ **MAUA** ]   [ **MAUB** ] , is formed by covalent cross linking of two tryptophan residues and incorporation of two oxygen atoms into one of the indole rings to form a quinone.  Ref: 10721104 Adv Exp Med Biol, 1999
- Methylamine dehydrogenase MADH  [ **MAUA** ]   [ **MAUB** ]  is a tryptophan tryptophylquinone TTQ dependent enzyme that catalyzes the oxidative deamination of primary amines.  Ref: 10985763 Biochemistry, 2000
- C H bond breakage by tryptophan tryptophylquinone TTQ dependent methylamine dehydrogenase MADH  [ **MAUA** ]   [ **MAUB** ]  occurs by vibrationally assisted tunneling Basran, J.  Ref: 11087744 J Biol Chem, 2001
- With regard to H transfer, studies with MADH  [ **MAUA** ]   [ **MAUB** ]  have provided the first example of ground state tunnelling of hydrogen driven by the natural, thermally activated, low frequency motions of the enzyme molecule.  Ref: 10830100 Biochem Soc Trans, 1999
- An alphaF55A MADH  [ **MAUA** ]   [ **MAUB** ]  PPy sensor was constructed, and its properties were compared to that of the native MADH  [ **MAUA** ]   [ **MAUB** ]  PPy sensor.  Ref: 11924976 Anal Chem, 2002
- Methylamine dehydrogenase MADH  [ **MAUA** ]   [ **MAUB** ]  and amicyanin form a physiologic complex which is required for interprotein electron transfer.  Ref: 10913294 Biochemistry, 2000
- Cyclopropylamine is a mechanism based inhibitor of the quinoprotein methylamine dehydrogenase MADH  [ **MAUA** ]   [ **MAUB** ]  from Paracoccus denitrificans.  Ref: 12062431 FEBS Lett, 2002
- To complete the catalytic cycle, MADH  [ **MAUA** ]   [ **MAUB** ]  is reoxidised via an electron transfer ET chain.  Ref: 12686162 Biochim Biophys Acta, 2003
- The Y119F and D32N mutants were purified from cell extracts and found to be significantly less stable than wild type MADH  [ **MAUA** ]   [ **MAUB** ] .  Ref: 11707614 Protein Eng, 2001
- Two putative monovalent cation binding sites in MADH  [ **MAUA** ]   [ **MAUB** ]  have been identified by X ray crystallography Labesse, G.  Ref: 11591147 Biochemistry, 2001
- Using site directed mutagenesis, phenylalanine 55 on the alpha subunit of MADH  [ **MAUA** ]   [ **MAUB** ]  was converted to alanine.  Ref: 11924976 Anal Chem, 2002
- Thus, it has been possible to create forms of MADH  [ **MAUA** ]   [ **MAUB** ]  that exhibit a preference for amines with carbon chain lengths of one, three, five, or seven carbons.  Ref: 11733518 J Biol Chem, 2002
- The PPy film containing MADH  [ **MAUA** ]   [ **MAUB** ]  was formed electrochemically on a gold minielectrode 1 mm diameter in the presence of ferricyanide.  Ref: 10845365 Anal Chem, 2000
- For L85, a hypertropic madH  [ **MAUA** ]   [ **MAUB** ]  mutant, the light growth response action spectrum is very similar to that of WT even though the photogravitropism action spectrum of L85 has been shown previously to be altered in the near UV region.  Ref: 11540649 Planta, 1991
- Methylamine dehydrogenase MADH  [ **MAUA** ]   [ **MAUB** ]  has been immobilized in a polpyrrole PPy film on an electrode surface and used as an amine sensor for the determination of primary amines.  Ref: 10845365 Anal Chem, 2000
- Only the T122S MADH  [ **MAUA** ]   [ **MAUB** ]  mutant was produced at near wild type levels.  Ref: 11707614 Protein Eng, 2001
- Relatively conservative mutations which removed the potentially reactive oxygens on the side chains of Thr122, Tyr119, Asp76 and Asp32 each resulted in greatly reduced or undetectable levels of MADH  [ **MAUA** ]   [ **MAUB** ]  production.  Ref: 11707614 Protein Eng, 2001
- Paracoccus denitrificans produces two primary enzymes for the amine oxidation, tryptophan tryptophylquinone TTQ containing methylamine dehydrogenase MADH  [ **MAUA** ]   [ **MAUB** ]  and quinohemoprotein amine dehydrogenase QH AmDH.  Ref: 12686147 Biochim Biophys Acta, 2003
- A model is also proposed to describe the sequence of events that leads to stable complex formation between MADH  [ **MAUA** ]   [ **MAUB** ]  and amicyanin.  Ref: 10913294 Biochemistry, 2000
- Possible roles for these amino acid residues in stabilizing unusual structural features of the MADH  [ **MAUA** ]   [ **MAUB** ]  beta subunit, protein folding and TTQ biosynthesis are discussed.  Ref: 11707614 Protein Eng, 2001
- Methylamine dehydrogenase MADH  [ **MAUA** ]   [ **MAUB** ]  is a tryptophan tryptophylquinone dependent enzyme that catalyzes the oxidative deamination of primary amines.  Ref: 11733518 J Biol Chem, 2002
- Methylamine dehydrogenase MADH  [ **MAUA** ]   [ **MAUB** ]  possesses an alpha 2 beta 2 structure with each smaller beta subunit possessing a tryptophan tryptophylquinone TTQ prosthetic group.  Ref: 12437349 Biochemistry, 2002
- The crystal structure of alphaF55A MADH  [ **MAUA** ]   [ **MAUB** ]  has also been determined with phenylhydrazine covalently bound to TTQ in the active site.  Ref: 12437349 Biochemistry, 2002
- Native MADH  [ **MAUA** ]   [ **MAUB** ]  exhibits a strong preference for methylamine over longer carbon chain amines.  Ref: 11733518 J Biol Chem, 2002
- Direct dynamics calculations employing hybrid quantum mechanical and molecular mehanical QM MM potentials and molecular dynamics simulation methods have been used to explore the important dynamic role that enzyme structure has on proton transfer in the C H bond breakage of a methylamine substrate by methylamine dehydrogenase MADH  [ **MAUA** ]   [ **MAUB** ] .  Ref: 12555860 Faraday Discuss, 2003
- Of the native and mutant proteins that were studied, their observed order of affinity for each other was as follows native MADH  [ **MAUA** ]   [ **MAUB** ]  and native amicyanin > native MADH  [ **MAUA** ]   [ **MAUB** ]  and R99D amicyanin > alpha D180R MADH  [ **MAUA** ]   [ **MAUB** ]  and native amicyanin > alpha D180R MADH  [ **MAUA** ]   [ **MAUB** ]  and R99D amicyanin, and alpha D180R MADH  [ **MAUA** ]   [ **MAUB** ]  and R99L amicyanin.  Ref: 10913294 Biochemistry, 2000
- The aauB and aauA genes encode the periplasmic aromatic amine dehydrogenase AADH large and small subunit polypeptides, respectively, and were homologous to  **mauB**  and  **mauA** , the genes for the large and small subunits of methylamine dehydrogenase MADH  [ **MAUA** ]   [ **MAUB** ] .  Ref: 11495996 Microbiology, 2001
- Methylamine dehydrogenase MADH  [ **MAUA** ]   [ **MAUB** ]  possesses an alpha 2 beta 2 subunit structure with each smaller beta subunit possessing a tryptophan tryptophylquinone TTQ prosthetic group.  Ref: 12686138 Biochim Biophys Acta, 2003
- No cross linking is seen with alphaF55A or alphaF55I MADH  [ **MAUA** ]   [ **MAUB** ]  mutants.  Ref: 12062431 FEBS Lett, 2002
- Site directed mutagenesis was used to alter active site residues of methylamine dehydrogenase MADH  [ **MAUA** ]   [ **MAUB** ]  from Paracoccus denitrificans.  Ref: 11707614 Protein Eng, 2001
- No activity or cross reactive protein was detected in extracts of cells expressing D76N, T122A and T122C MADH  [ **MAUA** ]   [ **MAUB** ]  mutants.  Ref: 11707614 Protein Eng, 2001
- Four residues of the beta subunit of MADH  [ **MAUA** ]   [ **MAUB** ]  which are in close proximity to the tryptophan tryptophylquinone TTQ prosthetic group were modified.  Ref: 11707614 Protein Eng, 2001
- The reduction of MADH  [ **MAUA** ]   [ **MAUB** ]  levels was determined by assays of activity and Western blots of crude extracts with antisera specific for the MADH  [ **MAUA** ]   [ **MAUB** ]  beta subunit.  Ref: 11707614 Protein Eng, 2001
- The immobilized MADH  [ **MAUA** ]   [ **MAUB** ]  on the electrode exhibited Michaelis Menten behavior similar to that of the free enzyme in solution with a Km value of 1.3 mM.  Ref: 10845365 Anal Chem, 2000
- When alphaPhe 55 is replaced with Ala, this preference is reversed with alphaF55A MADH  [ **MAUA** ]   [ **MAUB** ]  preferring long chain amines with at least seven carbons Zhu, Z.  Ref: 11733518 J Biol Chem, 2002
